# Cloning and characterization of the first serine carboxypeptidase from a plant parasitic nematode, *Radopholus similis*

**DOI:** 10.1038/s41598-017-05093-7

**Published:** 2017-07-06

**Authors:** Xin Huang, Chun-Ling Xu, Wan-Zhu Chen, Chun Chen, Hui Xie

**Affiliations:** Laboratory of Plant Nematology and Research Center of Nematodes of Plant Quarantine, Department of Plant Pathology, College of Agriculture, South China Agricultural University, Guangzhou, People’s Republic of China

## Abstract

*Radopholus similis* is an important parasitic nematode of plants. Serine carboxypeptidases (SCPs) are peptidases that hydrolyse peptides and proteins and play critical roles in the development, invasion, and pathogenesis of certain parasitic nematodes and other animal pathogens. In this study, we obtained the full-length sequence of the SCP gene from *R*. *similis* (*Rs*-*scp*-*1*), which is 1665 bp long and includes a 1461-bp open reading frames encoding 486 amino acids with an 18-aa signal peptide. This gene is a double-copy gene in *R*. *similis*. *Rs*-*scp*-*1* was expressed in the procorpus, esophageal glands and intestines of females and in the esophageal glands and intestines of juveniles. *Rs*-*scp*-*1* expression levels were highest in females, followed by juveniles and males, and lowest in eggs. *Rs*-*scp*-*1* expression levels were significantly suppressed after *R*. *similis* was soaked in *Rs*-*scp*-*1* dsRNA for 12 h. Nematodes were then inoculated into *Anthurium andraeanum* after RNAi treatment. Compared with water treatment, *R*. *similis* treated with RNAi were reduced in number and pathogenicity. In summary, we obtained the first SCP gene from a plant parasitic nematode and confirmed its role in the parasitic process.

## Introduction

The burrowing nematode *Radopholus similis* is a migratory endoparasitic plant nematode. *R*. *similis* was first discovered on banana roots from Fuji in 1891 and has since been found on more than 250 different plant species, including banana, citrus, black pepper, vegetables, ornamental plants and many crops that are important in global commerce. *R*. *similis* reportedly causes losses of 12.5 ton/ha in banana production worldwide^1^. There is currently no effective method to control this nematode. Therefore, it is particularly important to explore new approaches for *R*. *similis* control.

Proteases hydrolyse polypeptides or proteins, and there are many protease families, such as aspartic peptidases, cysteine peptidases, glutamic peptidases, metallopeptidases, asparagine peptide lyases, serine peptidases, mixed peptidases and threonine peptidases^2^. Hundreds of proteases have been found in plant parasitic nematodes^3^. Some of these proteases degrade plant cell walls or other defence-related proteins, making it easier for nematodes to migrate within plant tissues. Certain identified proteases are involved in nutrient digestion and development processes^4–7^.

Serine carboxypeptidases (SCPs) belong to the serine peptidase family, which is conserved in eukaryotes. These SCPs contain the ‘catalytic triad’ of Ser-Asp-His and an oxyanion hole^8^. In addition, these proteins contain the PROSITE serine carboxypeptidase motif, (LIVM)-X(GT)-E-S-Y-(AG)-(GS)^9^. SCPs were first described in baker’s yeast^10^. In recent years, SCPs have been shown to have a connection to body development and parasitism in some parasites and pathogens, such as *Sitodiplosis mosellana*, *Trypanosoma cruzi*, *Brugia malayi*, *Trichinella spiralis*, and *Angiostrongylus cantonensis* ^11–16^. Using proteomic methods, these proteins have also been detected in *Heligmosomoides polygyrus*, *Strongyloides ratti* and *Haemonchus contortus* ^17–19^. However, until now, SCPs have not been isolated from plant parasitic nematodes. In this study, the full-length sequence of *R*. *similis* SCP gene (*Rs*-*scp*-*1*) was amplified by performing RACE based on ESTs obtained from a previous study utilizing a suppression subtractive hybridization (SSH) library constructed from different pathogenic populations of *R*. *similis* ^20^. We investigated the expression and localization of *Rs*-*scp*-*1* by performing southern blotting, *in situ* hybridization and qPCR. We also clarified the role of *Rs*-*scp*-*1* in the reproduction and pathogenesis of *R*. *similis* using RNAi.

## Results

### *Rs*-*scp*-*1* sequence analysis

**Figure 1:**
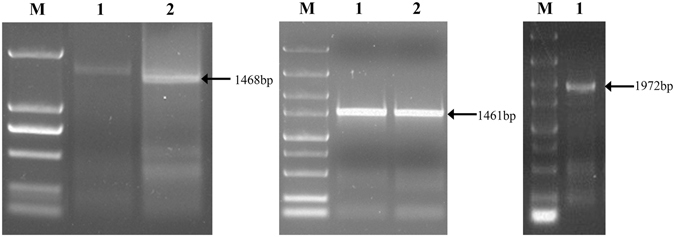
PCR amplification of *Radopholus similis Rs*-*scp*-*1*. (**A**) 5′ RACE amplification. M: DS2000 marker; 1: first-round RACE product; 2: nested PCR product. (**B**) ORF amplification. M, DS5000 marker; 1: product of *Rs*-*scp*-*1* ORF amplification. (**C**) *Rs*-*scp* gDNA amplification. M, DS5000 marker; 1: product of *Rs*-*scp*-*1* gDNA amplification.

**Figure 2:**
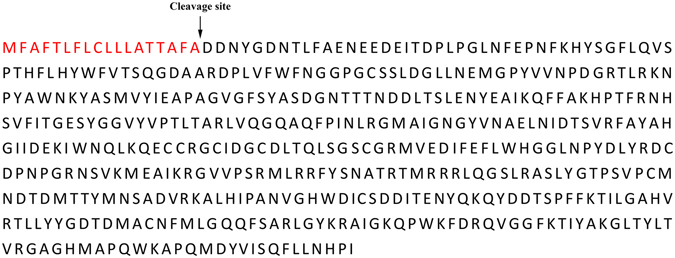
Sequence analysis of *Radopholus similis Rs*-*scp*-*1* (GenBank accession number: KJ617041.1). Signal peptide prediction of *Rs*-*scp*-*1*; signal peptide sequence is in red.

Based on ESTs obtained from a previously reported *R*. *similis* SSH library^20^, specific primers, specifically GSP and NEST-R, were employed to amplify the 5′ ends of *Rs*-*scp*-*1* (Fig. 1A). The *Rs*-*scp*-*1* cDNA consisted of a 69-bp 5′-untranslated region (5′-UTR), a 1461-bp ORF (KJ617041.1), and a 113-bp 3′-UTR (Fig. S1). *Rs*-*scp*-*1* was amplified with the gene-specific primers scp-F/scp-R (containing ATG and the stop codon, respectively) using cDNA and gDNA as templates (Fig. 1B,C). Introns were identified by aligning the genomic sequence to the corresponding cDNA sequence. *Rs*-*scp*-*1* contained 10 introns and 11 exons (Fig. S1). *Rs*-*scp*-*1* encoded a 486-amino acid sequence containing a peptidase S10 conserved domain and was 67% similar to *Caenorhabditis elegans* SCP. Sequence analysis indicated Rs-SCP-1 contains a signal peptide (Fig. 2) and may be secreted extracellularly.

**Figure 3:**
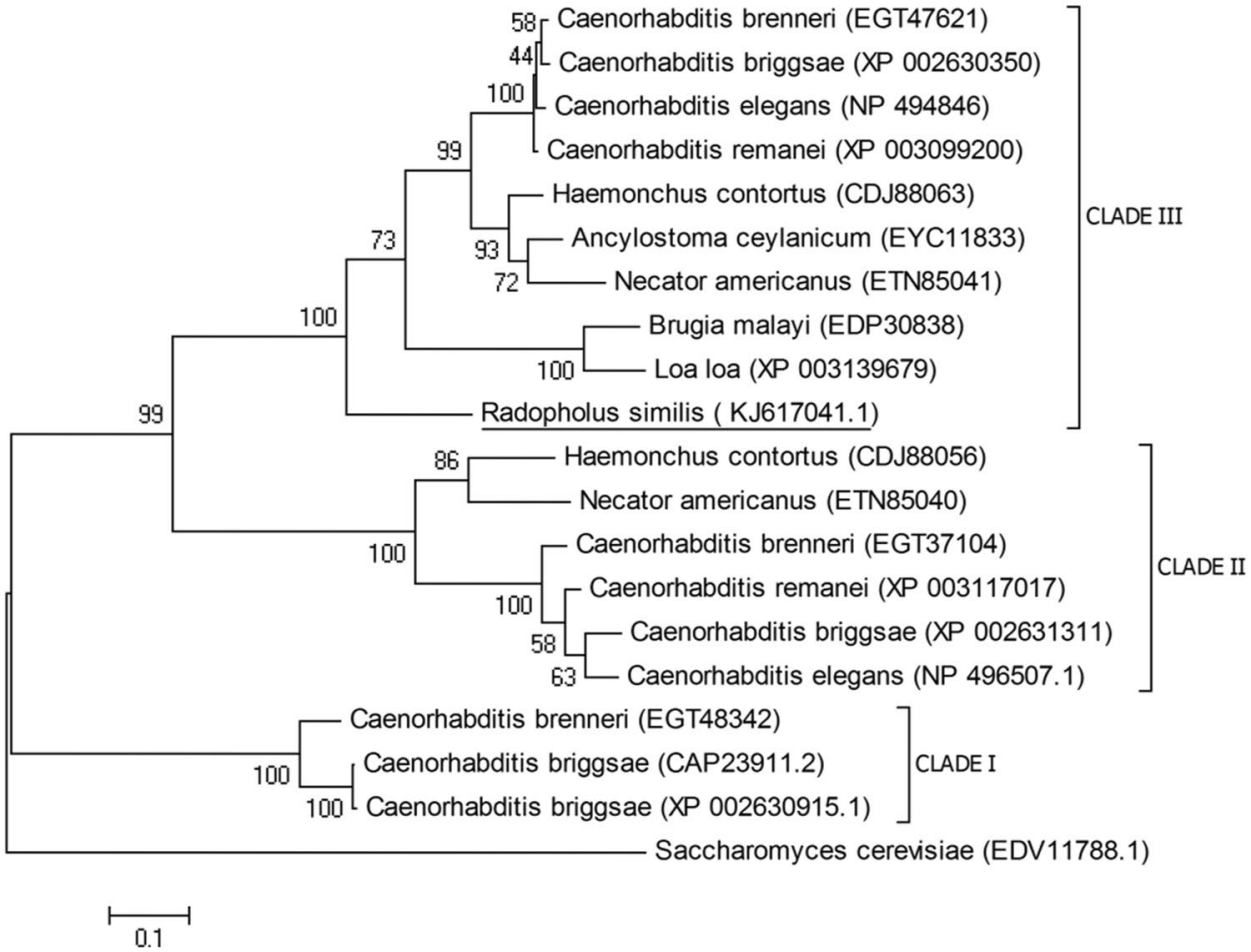
Maximum-likelihood phylogenetic tree containing 20 SCPs. A tree constructed with 20 SCPs from 10 species of nematodes and *Saccharomyces cerevisiae* was generated using MEGA 5. *Radopholus similis* SCP is underlined. Sequence accession numbers are shown in brackets.

A phylogenetic tree was constructed based on Rs-SCP-1 and 19 other SCP amino acid sequences (Fig. 3). All nematode SCPs were grouped into three branches. Rs-SCP-1 and SCPs of *C*. *brenneri* (GenBank: EGT47621), *C*. *briggsae* (XP 002630350), *C*. *elegans* (NP 494846), *C*. *remanei* (XP 003099200), *Haemonchus contortus* (CDJ88063), *Ancylostoma ceylanicum* (EYC11833), *Necator americanus* (ETN85041), *Brugia malayi* (EDP30838), and *Loa loa* (XP 003139679) were grouped on the same branch, suggesting they have a close phylogenetic relationship. However, Rs-SCP-1 was separated from these 9 SCP sequences. This separation may be attributable to the different lifestyle of these nematodes.

### Southern blot analysis

**Figure 4:**
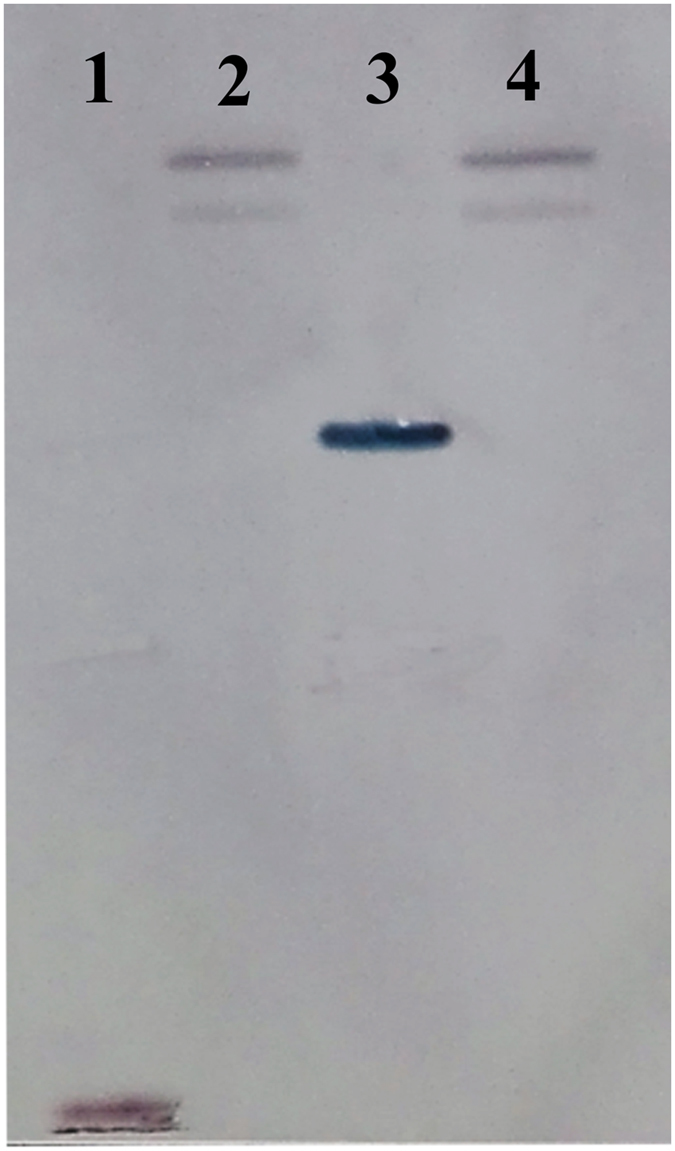
Southern blot analysis of *Radopholus similis Rs*-*scp*-*1*. 1, 3: pTA2-gscp digested with EcoR I and Hind III; 2, 4: *R*. *similis* gDNA digested with EcoR I and Hind III.

Southern blot analysis indicated the 489-bp-long, digoxigenin (DIG)-labelled probe hybridized to two fragments from *R*. *similis* gDNA digested with EcoR I and Hind III. Only one fragment was detected in pTA2-gscp digested with EcoR I and Hind III (Fig. 4). Based on these results, *Rs*-*scp*-*1* exists as a double-copy gene in the *R*. *similis* genome.

### Tissue localization and expression of *Rs*-*scp*-*1*

**Figure 5:**
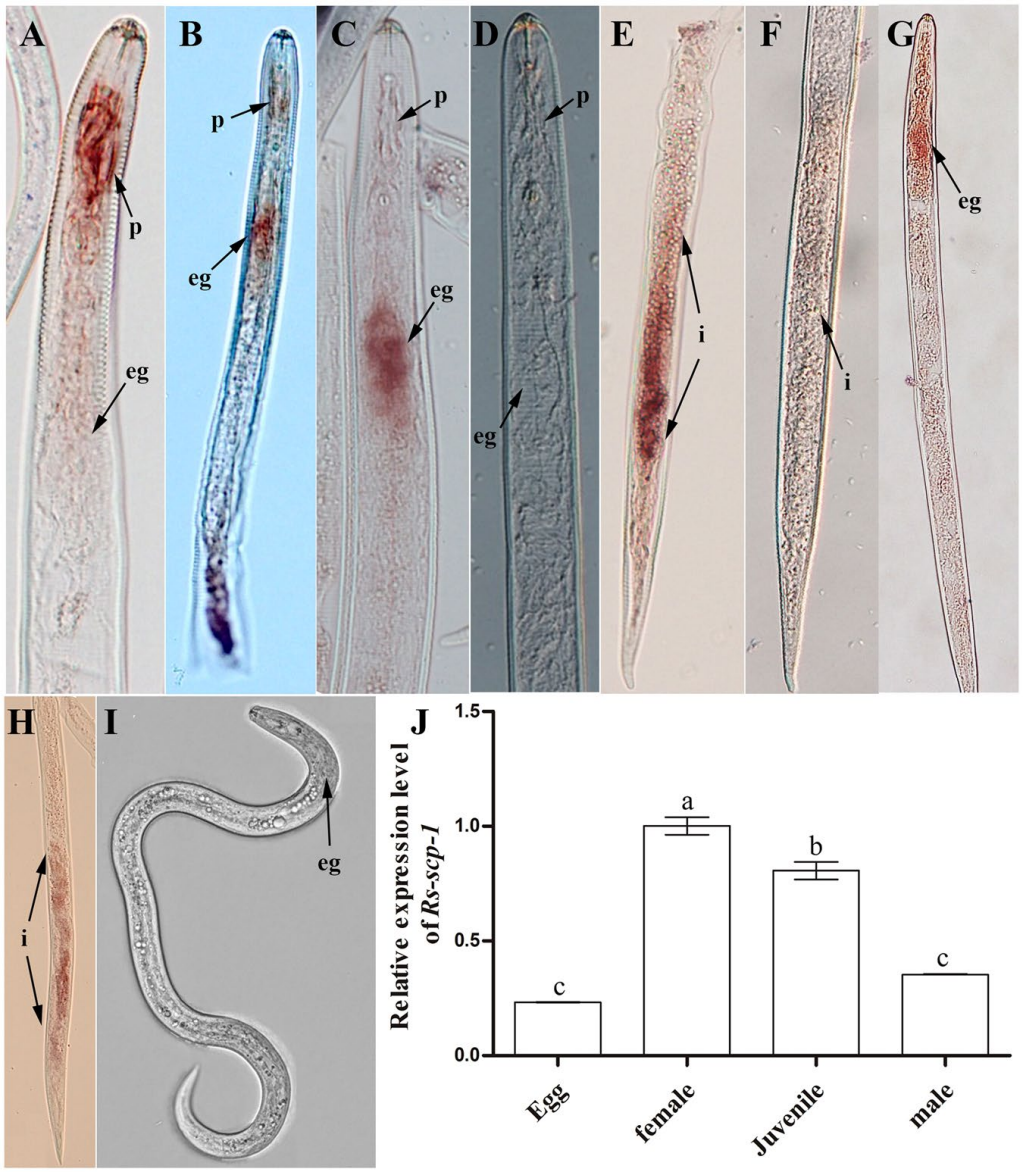
Tissue localization and expression levels of *Rs*-*scp*-*1* in *Radopholus similis*. (**A**–**I**) Tissue localization of *Rs*-*scp*-*1* mRNA *via in situ* hybridization. *Rs*-*scp*-*1* was located in the procorpus (**A**), oesophageal glands (**B**,**C**), and intestines (**E**) of females and the oesophageal glands (**G**) and intestines (**H**) of juveniles. There were no hybridization signals in control females (**D**,**F**) and juveniles (**I**) hybridized with a DIG-labelled sense *Rs*-*scp*-*1* RNA probe. p, procorpus; eg, esophageal glands; i, intestine. (**J**) *Rs*-*scp*-*1* expression levels at different life stages of *R*. *similis*. Bars indicate standard error of the mean (n = 3), and different letters indicate significant differences (p < 0.05) between treatments.

*In situ* hybridization results revealed the presence of *Rs*-*scp*-*1* mRNA in the procorpus (Fig. 5A), esophageal glands (Fig. 5B,C) and intestines (Fig. 5E) of females and in the esophageal glands and intestines (Fig. 5G,H) of juveniles. No hybridization signals were detected in females (Fig. 5D,F) and juveniles (Fig. 5I) after hybridization with a control sense probe. According to the qPCR results, *Rs*-*scp*-*1* expression was detectable at all life stages in *R*. *similis*. Expression in females was significantly higher (p < 0.05) than at other life stages. Expression in eggs, juveniles and males corresponded to 23%, 80%, and 35% of the expression observed in females, respectively (Fig. 5J). *Rs*-*scp*-*1* expression in juveniles was significantly higher (p < 0.05) than that observed in eggs and males. There was no significant difference (p > 0.05) in expression between eggs and males.

### RNAi and a pathogenicity test

**Figure 6:**
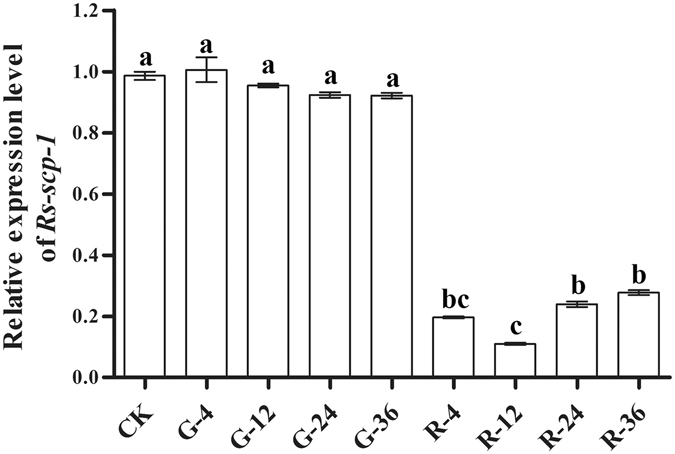
Expression of *Rs*-*scp*-*1* in *Radopholus similis* under different treatments. CK: blank control; G-4, G-12, G-24, and G-36: *R*. *similis* treated with *egfp* dsRNA for 4, 12, 24 and 36 h, respectively; R-4, R-12, R-24, and R-36: *R*. *similis* treated with *Rs*-*scp*-*1* dsRNA for 4, 12, 24 and 36 h, respectively. Bars indicate the standard error of the mean (n = 3), and different letters indicate significant differences (p < 0.05) between treatments.

qPCR was performed to detect *Rs*-*scp*-*1* expression in *R*. *similis* after dsRNA treatment for 4, 12, 24 or 36 h. Soaking in *Rs*-*scp*-*1* dsRNA solution led to a significant decrease (p < 0.05) in *Rs*-*scp*-*1* expression compared with controls. No significant difference (p > 0.05) was detected between untreated controls and nematodes treated with enhanced green fluorescent protein (*egfp*) dsRNA (Fig. 6). *Rs*-*scp*-*1* had the lowest expression levels among the treatments after 12 h of *Rs*-*scp*-*1* dsRNA soaking. There were significant differences (p < 0.05) between the 12-h versus 24- and 36-h treatments and no significant difference (p > 0.05) between the 12-h and 4-h treatments. Differences in expression levels also were not significant (p > 0.05) between the 4-h versus the 24- to 36-h treatments. Therefore, *Rs*-*scp*-*1* dsRNA soaked for 12 h achieved maximal silencing.

**Figure 7:**
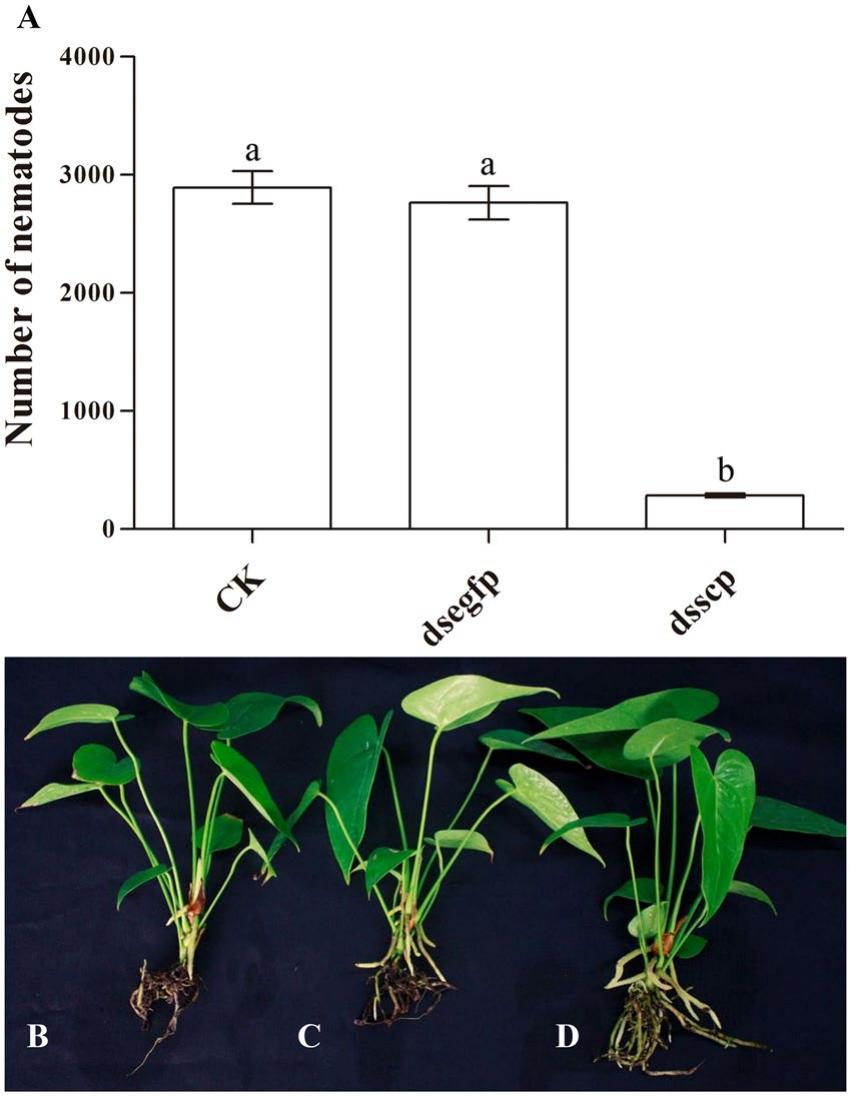
Effects of *Rs*-*scp*-*1* RNAi on the reproduction and pathogenicity of *Radopholus similis*. (**A**) Number of *R*. *similis* in anthurium roots. CK: untreated nematodes; dsegfp: nematodes treated with *egfp* dsRNA for 12 h; dsscp: nematodes treated with *Rs*-*scp*-*1* dsRNA for 12 h. Bars indicate the standard error of the mean (n = 5), and different letters indicate significant differences (p < 0.05) between treatments. (**B**) Anthurium inoculated with untreated nematode. (**C**) Anthurium inoculated with nematodes treated with *egfp* dsRNA for 12 h. (**D**) Anthurium inoculated with nematodes treated with *Rs*-*scp*-*1* dsRNA for 12 h.

Anthurium was inoculated with approximately 1,000 mixed-stage nematodes treated with *Rs*-*scp*-*1* dsRNA for 12 h. Sixty days after inoculation, nematode numbers in the roots were counted. Nematode numbers in roots inoculated with *Rs*-*scp*-*1* dsRNA-treated nematodes were significantly lower (284) (p < 0.05) than those observed in anthurium plants inoculated with untreated nematodes (blank control) (2,893) or nematodes treated with *egfp* dsRNA (2,763) (Fig. 7A). There was no significant difference (p > 0.05) in nematode numbers observed in plants inoculated with blank control nematodes and those inoculated with *egfp* dsRNA-treated nematodes. Only a few reddish-brown lesions were observed on anthurium plant roots inoculated with nematodes treated with *Rs*-*scp*-*1* dsRNA, whereas the roots on anthurium plants inoculated with untreated nematodes or nematodes treated with *egfp* dsRNA were largely rotted (Fig. 7B,C,D).

## Discussion

SCPs have a close relationship with parasitism in animal parasitic nematodes^15, 17–19^. The roles that SCPs play in plant parasitic nematodes are not yet clear. In this study, we cloned the SCP gene, *Rs*-*scp*-*1*, from *R*. *similis* and identified its structure and features. We confirmed the expression of *Rs*-*scp*-*1* mRNA in the procorpus, esophageal glands and intestines, and expression differs throughout the life cycle of *R*. *similis*. In addition, *Rs*-*scp*-*1* expression was significantly decreased after RNAi treatment, and *R*. *similis* pathogenicity in anthurium was significantly reduced after nematodes were treated with *Rs*-*scp*-*1* dsRNA for 12 h. This study is the first to investigate an SCP in a plant parasitic nematode.

SCPs are widespread in higher organisms and cleave C-terminal amino acid residues from peptides. Fifty-one proteins homologous to known SCPs have been found in the *Arabidopsis thaliana* genome. The SCP gene family encodes a diverse group of enzymes whose functions are likely to extend beyond protein degradation and processing to include activities such as the production of secondary metabolites^21^. Several SCPs have been found in nematodes, and some function in parasitism or development^14, 17, 18, 22^. According to a phylogenetic tree analysis, SCPs from different nematodes with different lifestyles separate into three branches by function^17, 22^. Therefore, there are likely to be several SCPs with different functions in *R*. *similis*. SCP is an excretory/secretory protein in *A*. *cantonensis* and *H*. *contortus* ^14, 17^. Rs-SCP-1 is phylogenetically related to the SCP in *H*. *contortus*. In this study, we confirmed that Rs-SCP-1 is an esophageal gland-secreted protein with a signal peptide in the N-terminus. *Rs*-*scp*-*1* was also detected in the procorpus of *R*. *similis*. This is consistent with the function of SCPs. Like other proteins that are secreted from the esophageal glands of plant parasitic nematodes, *Rs*-*scp*-*1* expressed in the esophageal glands of *R*. *similis* may help quickly destroy the host defence system and facilitate host invasion and the establishment of a parasitic relationship to obtain nutrients from the host^23–26^. In addition, *R*. *similis* pathogenicity decreased significantly after RNAi treatment. Thus, *Rs*-*scp*-*1* appears to be involved in *R*. *similis* parasitism. This gene may help *R*. *similis* degrade plant defence proteins, leading to higher *Rs*-*scp*-*1* expression levels in infective juveniles than in eggs and males. SCP in *B*. *malayi* belongs to the same branch as Rs-SCP-1 and is reportedly associated with developmental processes^22^. *Rs*-*scp*-*1* is located in the intestines of females and juveniles. Intestinal proteins related to nutritional absorption influence nematode development^6^. Thus, *Rs*-*scp*-*1* may be associated with nutrient uptake in the intestines of females and juveniles. *Rs*-*scp*-*1* mRNA hybridization signals were stronger and *Rs*-*scp*-*1* expression levels were higher in females than in juveniles. Meanwhile signal were not detected and *Rs*-*scp*-*1* expression was also significantly lower in males. These results were consistent with the biological functions of females, juveniles and males. Females and juveniles require more nutrition to complete development and infection processes. Females also bear the task of breeding and therefore need more nutrition than juveniles. As males are degenerate in the stylet and esophageal glands and have no ability to infect, their nutrient intake and absorption are weakened.

Based on the results of this study, *Rs*-*scp*-*1* may participate in nematode development and pathogenicity. The feasibility of targeting *Rs*-*scp*-*1* for the control of *R*. *similis* should be further investigated.

## Methods

### Nematode isolate

*R*. *similis*, isolated from *Zingiber officinale Roscoe* and cultured on carrot disks^27^, was employed in this study. Nematodes were extracted as described elsewhere^20^.

*Anthurium andraeanum* used in this study was purchased from the Flowers and Plants Research Center, Guangzhou, Guangdong. These plants were grown in a greenhouse at 26 ± 1 °C (16-h light/8-h dark photoperiod) and 60–80% relative humidity.

### DNA and RNA extraction

Approximately 20,000 mixed-stage nematodes cultured on carrot disks were collected for DNA extraction using phenol/chloroform^28^. The same number of nematodes was used for RNA extraction with TRIzol reagent (Invitrogen, Carlsbad, CA, USA). First-strand cDNA was synthesized using a RevertAid First Strand cDNA Synthesis Kit (Thermo Fisher, Waltham, MA, USA).

From the SSH library of *R*. *similis* ^20^, a fragment with a polyA structure was identified as a candidate for *Rs*-*scp*-*1* by performing a BLAST search of the NCBI database. 5′ RACE primers (GSP and NEST-R) (Table 1) were designed to amplify the 5′ end of *Rs*-*scp*-*1* using a SMART RACE cDNA Amplification Kit (Clontech, Takara Biotechnology (Dalian) Co., Ltd., Dalian, China). The two fragments of *Rs*-*scp*-*1* were then spliced into the complete sequence of *Rs*-*scp*-*1*. The ORF was predicted using ORFfinder (http://www.ncbi.nlm.nih.gov/gorf/orfig.cgi). Specific primers (scp-F/scp-R, Table 1) were designed to amplify the ORF sequence and genomic sequence of *Rs*-*scp*-*1*. These fragments were cloned into pTA2 for sequencing. The positive plasmids pTA2-scp and pTA2-gscp were stored at −20 °C for further use.

**Table 1:** Primer used in this study.

Primer name	Primer sequence
GSP	5′-TTCCTCCTCAGTCCTCACTCCTCAAA-3′
NEST-R	5′-GCGTACCGTCAAATAGGTCAATCC-3′
*scp*-F	5′-TTTGGCAATGTTCGCCTTCA-3′
*scp*-R	5′-TTCCTCCTCAGTCCTCACTCCTC-3′
*Rs*-*scp*T7-U	5′-TAATACGACTCACTATAGGGACAGTTCTTCGCCAAACACCC-3′
*Rs*-*scp*-D	5′-CCTCCATCTTGACCGAGTTCC-3′
*Rs*-*scp*-U	5′-ACAGTTCTTCGCCAAACACCC-3′
*Rs*-*scp*T7-D	5′-TAATACGACTCACTATAGGGCCTCCATCTTGACCGAGTTCC-3′
q179-F	5′-TGAATGTTAGAAACCCAATCAAAG-3′
q179-R	5′-CACTACGACACATTGAACCCCA-3′
Actin-F	5′-GAAAGAGGGCCGGAAGAG-3′
Actin-R	5′-AGATCGTCCGCGACATAAAG-3′
179i T7-F	5′-TAATACGACTCACTATAGGGAGCGATGACATCACCGAGA-3′
179i-R	5′-GGGTGGTTCAGCAGAAACT-3′
179i-F	5′-AGCGATGACATCACCGAGA-3′
179iT7-R	5′-TAATACGACTCACTATAGGGGGGTGGTTCAGCAGAAACT-3′
GFPiT7-F	5′-TAATACGACTCACTATAGGGTTCAAGTCCGCCATGCCCGAA-3′
GFPi-R	5′-CATGTGATCGCGCTTCTCGTT-3′
GFPi-F	5′-TTCAAGTCCGCCATGCCCGAA-3′
GFPiT7-R	5′-TAATACGACTCACTATAGGGCATGTGATCGCGCTTCTCGTT-3′
179SB-F	5′-GGAACACCACCACAAACGA-3′
179SB-R	5′-TGACGGGCAGACTGACCAT-3′

The T7 promoter sequence is underlined.

### Sequence analysis and phylogenetics

The sequence similarity of Rs-SCP-1 was analysed by performing a BLAST search of the NCBI non-redundant protein database (nr). Signal peptide and trans-membrane domains were predicted using SignalP 4.0 (http://www.cbs.dtu.dk/services/SignalP-4.0/) and TMHMM Server v. 2.0 (http://www.cbs.dtu.dk/services/TMHMM/), respectively. WoLF PSORT and PSORT II were used to predict protein subcellular localization. A phylogenetic tree containing SCPs from 11 species was constructed using the maximum-likelihood method.

### Southern blot analysis

The primers 179SB-F/179SB-R (Table 1) were designed to amplify a DIG-labelled probe using a PCR DIG Probe Synthesis Kit (Roche Applied Science, Penzberg, Germany). Approximately 10 μg of *R*. *similis* gDNA was digested with EcoR I and Hind III (Thermo Fisher) overnight at 37 °C. The digested DNA was separated via electrophoresis and transferred to a Hybond N^+^ membrane (Amersham Biosciences, GE Healthcare, UK). Hybridization and detection were performed using a Dig High Primer DNA Labeling and Detection Starter Kit I (Roche Applied Science) at 48 °C for 24 h. pTA2-gscp digested with EcoR I and Hind III was used as a control.

### mRNA *in situ* hybridization

The specific primers Rs-scpT7-U/Rs-scp-D and Rs-scp-U/Rs-scpT7-D (Table 1) were designed to amplify DIG-labelled sense and antisense probes using DIG RNA Labeling Mix (Roche Applied Science). *In situ* hybridization was performed as previously described^29^. After hybridization at 51.5 °C for 12 h, the nematodes were examined using Nikon Eclipse 90i microscope (Nikon, Kawasaki, Japan).

### Expression analysis of *Rs*-*scp*-*1*

RNA samples were extracted from 500 *R*. *similis* eggs, juveniles, females and males, respectively. Total RNA was then quantified with a NanoDrop spectrophotometer (Thermo Fisher). The RNA samples were used as templates for cDNA synthesis with HiScript II Q RT SuperMix for qPCR (Vazyme, Nanjing, China). Specific primers (q179-F/q179-R, Table 1) were designed to assay *Rs*-*scp*-*1* expression. The primers Actin-F/Actin-R (Table 1)^30^ were used to amplify β-actin as a reference gene. qPCR was performed using a CFX96 qPCR instrument (Bio-Rad, Hercules, CA, USA) with AceQ qPCR SYBR Green Master Mix (Vazyme). All expression experiments were performed in triplicate with three biological replicates.

### RNAi and silencing detection

The primers 179iT7-F/179i-R and 179i-F/179iT7-R (Table 1), which contained a T7 promoter, were designed to amplify *Rs*-*scp*-*1* sense and antisense single-stranded RNA (ssRNA) using a Script Max^TM^ Thermo T7 Transcription Kit (TOYOBO, Osaka, Japan). Equal amounts of sense and antisense ssRNA were mixed and incubated at 75 °C for 10 min to hybridize and form dsRNA. The dsRNA was purified with a 1/10 volume of NaAc (3 mol/L) and a 2-fold volume of ethanol at −20 °C overnight, washed twice with 70% ethanol, and then dissolved in deionized water. The quantity of dsRNA was measured using a NanoDrop spectrophotometer and analysed by performing gel electrophoresis in a 1.2% agarose gel. Finally, the dsRNA was stored at −80 °C for later use. Non-endogenous control *egfp* dsRNA was synthesized using the primers GFPiT7-F/GFPi-R and GFPi-F/GFPiT7-R (Table 1).

Approximately 500 mixed-stage nematodes were soaked in *Rs*-*scp*-*1* dsRNA solution (2.0 μg/μL) for 4, 12, 24, or 36 h. Nematodes soaked in *egfp* dsRNA solution were used as non-endogenous controls. The soaking times for the controls were the same as those for the *Rs*-*scp*-*1* dsRNA. In addition, untreated nematodes were used as blank controls. Nematodes treated with dsRNA were washed three times with ddH_2_O, and then RNA was extracted and qPCR was performed using the methods described above to analyse the suppression of *Rs*-*scp*-*1* mRNA expression in *R*. *similis*.

Approximately 1,000 mixed-stage nematodes treated with *Rs*-*scp*-*1* dsRNA were employed for pathogenicity analysis. Untreated nematodes and those treated with *egfp* dsRNA solution were used as controls. Sixty days after inoculation, rhizosphere nematodes were isolated and counted. Plant inoculation and nematode counting were performed based on previously described methods^20^. Five biological replicates were performed per trial, and the experiment was performed twice.

### Data analysis

Statistical analysis was performed using SPSS 23.0. All data in this study were subjected to analysis by one-way ANOVA and tested for differences between treatments at a 5% level using Duncan’s Multiple Range Test (DMRT).

## Electronic supplementary material

Supplementary Information
