# Supplementary material for: Cloning and characterization of the first serine carboxypeptidase from a plant parasitic nematode, Radopholus similis
Source: Sci Rep. 2017 Jul 6;7:4815. doi: 10.1038/s41598-017-05093-7 (PMC5500496; doi:10.1038/s41598-017-05093-7)
Supplement: Supplementary file 1 — Supplementary Information [file 41598_2017_5093_MOESM1_ESM.pdf]

Cloning and characterization of the first serine carboxypeptidase from a plant parasitic nematode, *Radopholus similis*

Xin Huang[1], Chun-Ling Xu[1], Wan-Zhu Chen[1], Chun Chen[1], Hui Xie[1*]

[1]Laboratory of Plant Nematology and Research Center of Nematodes of Plant Quarantine, Department of Plant Pathology, College of Agriculture, South China Agricultural University, Guangzhou, People's Republic of China.

[*]correspondence and requests for materials should be addressed to H.X. (email: xiehui@scau.edu.cn)

```
1     aagcagtggt atcaacgcag agtacatggg gaccttcact tttcgccatt atcgcgttcc
61    catttggcaA TGTTCGCCTT CACACTTTTT CTATGTCTCC TCCTCGCTAC CACTGCGTTT
121   GCTGATGACA ACTATGGGGA CAACACGTTG TTCGCGGAGA ACGAGGAGGA CGAGATCACT
181   GACCCACTGC CCGGACTCAA CTTCGAACCG AACTTCAAGC ATTACTCAGG TGGCCTGTAG
241   CACATCCGCA GATTAGGTAT ATACCTTCTT TCGATTTTCA GGATTCCTCC AAGTATCGCC
301   CACCCACTTT TTGCACTACT GGTTTGTCAC CTCGCAAGGA GATGCGGCAC GTGATCCGCT
361   GGTCCGCTAA CACTTCCCTT TTACTTTGTT AAATTTTTTA TTCAGGTGTT CTGGTTCAAC
421   GGTGGTCCGG GCTGTTCGTC GCTGGACGGG CTCCTGAACG AGATGGGTCC ATACGTGGTG
481   AATCCGGATG GCCGCACTTT GCGCAAGAAT CCGTACGCAT GGAACAAATA CGCGTCCATG
541   GTGTACATCG AGGCTCCGGC TGGCGTTGGA TTCTCGTACG CGTCTGACGG GAACACCACC
601   ACAAACGACG ACCTGGTCTC TTTCGGAGGA GCAGAATGGA TCTATTGTAG ACATTTTGCC
661   TTCAGACCTC TTTGGAGAAT TACGAGGCGA TCAAACAGTT CTTCGCCAAA CACCCCACAT
721   TCCGCAACCA TTCCGTCTTC ATCACGGGCG AATCGTACGG AGGCGTGTAC GTGCCAACCC
781   TCACCGCCCG ATTGGTGCAG GGACAGGCAC AATTCCCGAT CAACCTGAGG GGCATGGCTA
841   TCGGCAACGG ATACGTGAAT GCCGAGCTAA ACATCGACAC GTCGGTCCGA TTCGCGTACG
901   CGCACGGCAT CATCGACGAA AAGATCTGGA ACCAACTGAA GCAAGAGTGC TGTCGTGGGT
961   GCATTGGTAG GGGAATCGGG TGGGAATCAA CTTGCCGTTG TCATTGCCTC ACATTCAGAT
1021  GGTTGCGACC TGACTCAGCT GAGTGGCTCC TGCGGACGAA TGGTCAGTCT GCCCGTCACA
1081  GTTTGGGAGC ACTTGCATAC CCCCGCTATT TAGGTGGAGG ACATTTTTGA ATTCCTCTGG
1141  CACGGGGGCC TCAACCCATA CGACCTGTAC CGCGACTGCG ATCCCAATCC GGGCAGGAAC
1201  TCGGTCAAGA TGGAGGCCAT CAAGAGGGGC GTCGTGCCTA GTCGGATGCT GCGTCGCTTC
1261  TACTCCAACG CGACGCGAAC AATGCGCCGA CGCCTGCAAG TGAGTTGGCC CAGAAAATGG
1321  GGACCACGGC ATTCCATTTG AGGGATCATT GCGCGCCTCG CTGTACGGAA CCCCTTCAGT
1381  GCCGTGCATG AACGGTATGC ATCGGAGAGA CTTCGAAAAG GAGGGAGGGG TGCCCCTTCA
1441  GACACCGACA TGACCACGTA CATGAACAGT GCCGATGTGC GCAAGGCGCT GCACATTCCC
1501  GCCAATGTGG GCCACTGGGA CATTTGCAGG TAGGGAGGGG AGAGAAGGAA CCACCGAATC
1561  GGGGAACTGT TTGTTTAGCG ATGACATCAC CGAGAACTAC CAGAAGCAAT ACGGCAAGTG
1621  TCGCGAGCAG ATGTCGTACA AAAGCATGTC CTACTTGTTT CAGACGACAC GTCGCCATTC
1681  TTCAAGACCA TTCTGGGCGC TCATGTGCGC ACTCTGCTCT ACTACGGGGA CACGGACATG
1741  GCGTGCAACT TCATGCTGGG GCAGCAGTTT TCCGCCAGAC TGGGCTACAA ACGCGCCATC
1801  GGAAAACAGC CGTGGAAATT CGACCGCCAA GTCGGCGGAT TCAAAACCAT CTACGCGAAG
1861  GGATTGACCT ATTTGACGGG TAAAGTGGGG GAGGAAACAA CAATAAAACT CTGTTCTCAC
1921  TCAGTGCGCG GAGCGGGGCA CATGGCGCCG CAGTGGAAGG CACCGCAAAT GGACTACGTG
1981  ATCAGCCAGT TTCTGCTGAA CCACCCTATT TGAggagtga ggactgagga ggaaggaaat
2041  agtgtcaacc cccagttatg ctatctactt gccgatgagc gcataattaa gcatctgtaa
2101  aaaaaaaaaa aaaaaaaaaa aaaaaa
```

Fig. S1 Genomic sequence of *Radopholus similis Rs-scp-1*.

The *Rs-scp-1* genomic coding region contains 10 introns and 11 exons. Introns are marked in grey. ATG, initiation codon; TGA, stop codon.
